# Quantifying uncertainty in graph neural network explanations

**DOI:** 10.3389/fdata.2024.1392662

**Published:** 2024-05-09

**Authors:** Junji Jiang, Chen Ling, Hongyi Li, Guangji Bai, Xujiang Zhao, Liang Zhao

**Affiliations:** ^1^School of Management, Fudan University, Shanghai, China; ^2^Department of Computer Science, Emory University, Atlanta, GA, United States; ^3^School of Computer Science and Technology, Xidian University, Shanxi, China; ^4^Data Science & System Security, NEC Labs America, Princeton, NJ, United States

**Keywords:** uncertainty quantification, graph neural network, variational mechanism, explanation uncertainty, deep learning

## Abstract

In recent years, analyzing the explanation for the prediction of Graph Neural Networks (GNNs) has attracted increasing attention. Despite this progress, most existing methods do not adequately consider the inherent uncertainties stemming from the randomness of model parameters and graph data, which may lead to overconfidence and misguiding explanations. However, it is challenging for most of GNN explanation methods to quantify these uncertainties since they obtain the prediction explanation in a *post-hoc* and model-agnostic manner without considering the randomness of *graph data* and *model parameters*. To address the above problems, this paper proposes a novel uncertainty quantification framework for GNN explanations. For mitigating the randomness of graph data in the explanation, our framework accounts for two distinct data uncertainties, allowing for a direct assessment of the uncertainty in GNN explanations. For mitigating the randomness of learned model parameters, our method learns the parameter distribution directly from the data, obviating the need for assumptions about specific distributions. Moreover, the explanation uncertainty within model parameters is also quantified based on the learned parameter distributions. This holistic approach can integrate with any *post-hoc* GNN explanation methods. Empirical results from our study show that our proposed method sets a new standard for GNN explanation performance across diverse real-world graph benchmarks.

## 1 Introduction

Explaining the prediction of deep graph models, e.g., Graph Neural Networks (GNNs), is crucial for enhancing the model interpretability and trustworthiness of its prediction, which has played a crucial role in various domains. For instance, in drug discovery, GNNs can model molecular structures and interactions to identify potential drug candidates. By explaining the prediction results, researchers can gain insights into the molecular properties that drive drug effectiveness and safety for further improving the design (Mastropietro et al., 2022). In social network analysis, GNN explanations can help analyze user behaviors, preferences, and relationships in social networks. This information can be used to improve user experience, detect malicious activities, and develop targeted marketing strategies (Ying et al., 2019).

Existing techniques tend to explain the prediction of GNNs at the instance level. These approaches (Pope et al., 2019; Ying et al., 2019; Schnake et al., 2021) focus on identifying the importance of individual elements from the input graph, such as nodes (or node features), subgraphs, or edges that have a substantial impact on the predicted labels, based on taking the gradient of the output with respect to the input, effectively showing how much the output would change with small alteration of input. While these techniques offer valuable insights into the decision-making process of GNNs, these methods generally overlook the potential uncertainty that may exist in the generated explanations. *Uncertainty in explanations* can stem from various sources, including the inherent noise in the input graph, model parameter uncertainty, and the approximation techniques used in the explanation methods themselves. *Post-hoc* explanations tend to be sensitive to these uncertainties, and neglecting this uncertainty can lead to overconfidence in the generated explanations, potentially resulting in misguided decision-making in critical applications where the stakes are high. For instance, a pharmaceutical company may use GNNs to identify the most critical features of the input protein graph, such as specific molecular substructures, contributing to the targeted biology activities. If the GNN explanation method neglects the uncertainty present in the generated explanation, the company may be overconfident in the identified critical features without considering alternative explanations, which may lead to suboptimal real-world efficacy or unforeseen side effects. Therefore, considering the uncertainty into explainable GNNs would allow users to better assess the reliability of the explanations, ultimately increasing their confidence in making decisions for real-world applications.

However, quantifying the explanation uncertainty of GNN is not a trivial task, and current uncertainty quantification methods designed for GNNs cannot be simply adapted for two primary obstacles. The first pertains to *the difficulties of quantifying uncertainties in explanations resulting from the intrinsic randomness of graph data*. Specifically, there is an inherent variability in the attributes connected to the nodes and edges within the graph. For instance, node features might contain unexpected noise during measurement or yield an unsuitable node permutation. Furthermore, the graph connectivity may also be uncertain, which can result from missing nodes or edges, fluctuations in the graph topology over time, or noise within the graph structure itself. Such factors can introduce uncertainty into both the model's predictions and the explanations thereof. However, existing uncertainty quantification methods for GNNs, as illustrated by Zhao et al. (2020); Munikoti et al. (2023), are unable to account for these complexities in the GNN prediction explanation, rendering their simple adaptations to quantify the explanation uncertainty on graph data unfeasible. The second obstacle involves *the difficulty of quantifying explanation uncertainty without making assumptions about the distribution of model parameters*. GNNs, similar to other deep learning models, are subject to uncertainties regarding the network parameters and architectures that optimally represent the underlying graph data. Providing explanations of model predictions without acknowledging this uncertainty could lead to overconfidence in interpretation, particularly when the model has not been trained on data that is representative of the task. Current uncertainty quantification methods for GNNs are not readily adaptable for quantifying the explanation uncertainty brought by model parameters, as they generally assume a predefined (e.g., Gaussian) distribution that the model parameters are expected to follow. However, the majority of techniques for explaining GNNs are *post-hoc*, implying that they are implemented after the model training. These techniques endeavor to elucidate the model's predictions based on its learned parameters, yet they do not explicitly account for the uncertainty present in these explanations. In this context, to quantify the explanation uncertainty instigated by model parameters, it is not feasible to make distribution assumptions about model parameters.

In light of two predominant challenges, we introduce EU-GNN, a novel and adept framework specifically designed to quantify the uncertainty of explanations in graph classification. To meticulously address the inherent randomness embedded within graph data, we propose two distinctive and unique data uncertainties related to graphs. These carefully formulated uncertainties are strategically utilized to directly and precisely quantify the uncertainty present in GNN explanations, an uncertainty that is induced by both of these data uncertainties. When it comes to managing the parameter distribution randomness, our approach astutely takes a slight, but impactful, departure from conventional methods, which often rely heavily on prescribed distributions. Instead, we introduce an innovative end-to-end framework that learns the parameter distribution directly from the data, thereby circumventing the need to make presumptions about specific distributions. It's noteworthy that this framework, with its intuitive design, can be seamlessly integrated with most *post-hoc* GNN explanation techniques, thereby significantly enhancing their ability to provide both reliable and uncertainty-aware explanations of GNN predictions. This dual-pronged approach, not only addresses but ingeniously navigates through the aforementioned challenges, offering a comprehensive and thorough solution for quantifying explanation uncertainty in GNNs. Our main contributions are summarily encapsulated as follows.

- **Problem**. We formulate the problem of quantifying the uncertainty in explanations of GNN prediction from the perspective of the different inherent randomness of the graph data and model parameters.
- **Method**. We identify two types of data uncertainty originating from graph data and a novel way to quantify parameter uncertainty. Specifically, we propose an end-to-end framework to model the parameter distribution from a generative learning perspective without making distribution assumptions about model parameters. Meanwhile, the framework supports most *post-hoc* GNN explanation techniques to provide reliable explanations with uncertainty quantification.
- **Experiment**. We conduct extensive experiments on three molecule classification datasets. Compared with state-of-the-art baselines, EU-GNN achieves the best performance on both graph classification and misclassification detection. In addition, EU-GNN is proven to be effective in quantifying the various uncertainties of explanation.

## 2 Related work

### 2.1 Uncertainty quantification in graph neural networks

Uncertainty Quantification (Ling et al., 2024) aims to provide a reliable estimation of uncertainties associated with data and model predictions, which is crucial for decision-making (Ling et al., 2022, 2023a). Gal and Ghahramani (2016) first introduced dropout as a Bayesian approximation to model uncertainty in deep learning, providing an efficient and scalable framework for uncertainty estimation in neural networks. Several recent studies have focused on developing novel techniques for efficient and accurate UQ, including Bayesian inference (Mobiny et al., 2021), ensemble methods (Wen et al., 2020), and the single deterministic network containing explicit components to represent aleatoric and epistemic uncertainty (Raghu et al., 2019). With the development of GNNs, rising attention has been focused on the field of UQ for GNNs. Zhang et al. (2019) treat observed graphs as realizations from a parametric family, jointly inferring network structure and GNN parameters, enhancing model robustness. Pal et al. (2019) build upon this, using the MAP estimate of the graph for learning tasks, capturing aleatoric uncertainty through the mode of the graph structure's probability density function. Munikoti et al. (2023) propose a unified Bayesian approach, employing Assumed Density Filtering for aleatoric uncertainty and Monte Carlo dropout for model parameter uncertainty.

### 2.2 Explainable graph neural network

GNNs have attracted considerable attention in terms of interpretability. The mainstream GNN explanation methods can be broadly classified into four categories. First, gradient-based methods utilize gradients as indicators of the importance of different input features. Prominent examples of such methods include Guided Backpropagation (Guided BP) (Baldassarre and Azizpour, 2019) and Gradient-weighted Class Activation Mapping (Grad-CAM) (Pope et al., 2019). The second category comprises perturbation-based methods, which typically involve an additional optimization step to identify the most influential inputs by perturbing them. GNNExplainer (Ying et al., 2019) and GraphMask (Schlichtkrull et al., 2020) are notable methods in this category. Response-based methods constitute the third category, wherein the output response signal is backpropagated as an importance score layer-by-layer until it reaches the input space. Representative methods include Layer-wise Relevance Propagation (LRP) (Baldassarre and Azizpour, 2019) and GNNLRP (Schnake et al., 2021). The fourth category encompasses surrogate-based methods, which explain the original model by deriving explanations from an interpretable surrogate model trained to approximate the original model's predictions, including GraphLIME (Huang et al., 2022), RelEx (Schnake et al., 2021), and PGM-Explainer (Vu and Thai, 2020).

## 3 Methodology

In this section, we propose the problem formulation along with a novel concept of explanation uncertainty, which is derived from two types of uncertainties in the graph classification task. We first introduce how we can derive explanation uncertainty based on the variance from graph data. We then introduce an end-to-end framework to quantify the explanation uncertainty by considering the inherent randomness of learned parameters from the variational inference's perspective.

### 3.1 Problem formulation

Consider a graph *G* = (*V, E, X, A*) consisting of a set of nodes *V* and a set of edges *E*⊆|*V*| × |*V*|. Each node *v*_*i*_∈*V* consists of a feature $x_{i}\in {\mathbb{R}}^{d_{in}}$, and the feature set is represented as $X\in {\mathbb{R}}^{|V|\times d_{in}}$, where *d*_*in*_ is the dimension of raw node features. The connectivity of *G* is recorded in an adjacency matrix *A*∈{0, 1}^|*V*| × |*V*|^. *A*_*ij*_ = 1 if there is an edge between node *i* and *j*, otherwise *A*_*ij*_ = 0 denotes the edge does not exist. The graph classification aims at learning a mapping function G↦c,c∈C that maps *G* to a class *c* from a set of labels C, where the mapping function is typically a Graph Neural Network.

#### 3.1.1 Aleatoric uncertainty on graph classification

AU refers to the intrinsic randomness of the graph data, which can further be attributed to inaccurate measurements of node features (i.e., measurement uncertainty) or volatile graph structures (i.e., structural uncertainty). Specifically, *(1) Measurement Uncertainty* refers to the associated error in the node features, i.e., the observed node feature *X* is considered as the sum of true feature $\tilde{X}$ and a measurement error ε sampled from a latent distribution *p*(ε). *(2) Structural Uncertainty* is associated with the probabilities of link existence. Intuitively, the observed *E* may not reflect the true connectivity between nodes. In the social network scenario, User A and B are friends; User B and C are also friends. Then, Users A and C are likely to be friends, but the connectivity may not be reflected in the existing observation. It is worth noting that both measurement and structural uncertainty do not impact the graph label *c* since these small deviations may not significantly affect the broader patterns and structures that the graph *G* conveys (Munikoti et al., 2023). However, the explanation of the GNN prediction could be impacted due to these deviations.

#### 3.1.2 Epistemic uncertainty on graph classification

EU is the scientific uncertainty in the model that exists because of model in-competency to completely explain the underlying process. Let F(·) be an *L*-layer graph classification neural network with the trainable parameters $\Omega ={\left\{\omega_{l}\right\}}_{l=1}^{L}$, where ω_*l*_ is the parameter for the *l*-th layer. The epistemic uncertainty in the context of GNN is referred to the parametric uncertainty. Specifically, the parameters Ω of the GNN model F(·) are assumed to be probabilistic with a probability density function *p*(Ω).

#### 3.1.3 *Post-hoc* explanation generation

To highlight components (e.g., nodes and edges) that contribute significantly to the model's final decision, existing methods typically generate the importance based on the learned GNN parameters Ω in a *post-hoc* way. Specifically, we differentiate the output of the model with respect to the model input, thus creating a heat-map (i.e., saliency map), where the norm of the gradient over input variables indicates their relative importance. Such a saliency map corresponding to class *c* is denoted by *S*^*c*^ = *g*(*X, A*, Ω), where *g*(·) is the explanation generation function, e.g., Grad-CAM (Pope et al., 2019). The shape of *S*^*c*^ may vary depending on the specific component (nodes or edges) that is intended to be emphasized. For a node-level saliency map, *S*^*c*^∈ℝ^|*V*|^. For an edge-level saliency map, its dimension is ℝ^|*V*| × |*V*|^.

In this work, we focus on the problem of Uncertainty Quantification in explaining graph learning tasks, especially on graph classification, which involves obtaining the *variance* in the graph classification predictions and corresponding explanations caused by both aleatoric and epistemic uncertainties. However, quantifying the explanation uncertainty entails solving two critical challenges. First, the volatility of both *X* and *A* may propagate through the layers of the GNN and ultimately affect the model prediction as well as the corresponding explanation. Existing methods lack a clear formulation to quantify the explanation uncertainty caused by different aleatoric uncertainties. Second, most explanation generation methods are *post-hoc* and do not involve in training Ω. However, existing uncertainty quantification methods tend to assume an underlying distribution (e.g., Gaussian Distribution) that the *p*(Ω) may follow in order to address the epistemic uncertainty. In this scenario, it is impractical to quantify explanation uncertainty caused by model parameters by making distribution assumptions about them.

### 3.2 Explanation uncertainty derivation from the variance of graph data

Aleatoric (Data) Uncertainty often occurs when there is a measurement error ε or unobserved connections between the observed graph *G* and the true graph $\tilde{G}$. To quantify the explanation uncertainty brought by the aleatoric uncertainty, we introduce a graph acquisition function to augment the observed graph data in order to allow the GNN to encounter a diverse range of uncertain graph data during both learning and inference phases. Specifically,


(1) $$\begin{array}{l} G=T_{\xi }\left(\tilde{G}\right)+\varepsilon , \end{array}$$

where T is a reversible transformation operator that is applied to *G*. ξ is the set of parameters of the transformation, and ε represents the noise that is added to the transformed graph. For node features, the reversible transformation operator adds white noises. For the adjacency matrix, we permute the order of nodes as the transformation operator. Note that the reversible transformation would not change the graph. We subsequently introduce different ways of quantifying the Explanation Uncertainty from the *Measurement Uncertainty* and the *Structural Uncertainty*, respectively.

#### 3.2.1 Explanation uncertainty from measurement uncertainty

According to Equation 1, the measurement uncertainty exists when we encounter deviation in measuring the node features or getting an inappropriate node permutation. To reversely obtain the original graph instance, we have $\tilde{G}=T_{\xi }^{-1}\left(G-\epsilon \right)$. Based on the calculation process of the saliency map, we also have ${\tilde{S}}^{c}=g\left(\tilde{X},Ã,\Omega \right)$. Note that the corresponding saliency map changes during the test phase when the input graph *G* is transformed. However, the predicted classification label should remain unchanged, i.e., *y* = ỹ.

We aim to infer the explanation of the classification result of the original graph $\tilde{G}$:


$$\begin{array}{l} S^{c}=T_{\xi }({\tilde{S}}^{c})=T_{\xi }(g(\tilde{X},\tilde{A},\Omega ))=T_{\xi }(g(T_{\xi }^{-1}(X-\varepsilon ),\tilde{A},\Omega )). \end{array}$$

Note that we omit the subscripts and denote the saliency map obtained at the last GNN layer $S_{L}^{c}$ as *S*^*c*^ for the sake of simplicity. The distribution of the explanation given the input graph *G* is:


$$\begin{array}{l} p(S^{c}|G)=p(T_{\xi }(g(T_{\xi }^{-1}(X-\varepsilon ),\tilde{A},\Omega ))),\varepsilon \sim p(\varepsilon ),\xi \sim p(\xi ). \end{array}$$

The final prediction of the explanation is computed by the expectation of *S*^*c*^:


(2) $$\begin{array}{l} {𝔼}_{S^{c}\sim p\left(S^{c}|G\right)}[S^{c}]=\int S^{c}p\left(S^{c}|G\right)dS^{c}\\ \;=\iint T_{\xi }\left(g\left(T_{\xi }^{-1}\left(X-\varepsilon \right),\tilde{A},\Omega \right)\right)p\left(\varepsilon \right)p\left(\xi \right)\;d\varepsilon d\xi . \end{array}$$

The integration in Equation (2) can be approximated by Monte Carlo integration. In the *i*-th simulation run, we first sample a noise ε_*i*_ from the prior distribution, then compute the *i*-th explanation inference by $S_{i}^{c}=T_{\xi }\left(g\left(T_{\xi }^{-1}\left(X-\varepsilon_{i}\right),Ã,\Omega \right)\right)$. The expectation is approximated by ${𝔼}_{S^{c}\sim p\left(S^{c}|G\right)}\left[S^{c}\right]\approx \frac{1}{I}\overset{I}{\underset{i=1}{\sum }}S_{i}^{c}$. Finally, the variance of explanation caused by the measurement uncertainty is computed as:


$$\begin{array}{l} \frac{1}{I-1}\sum_{i=1}^{I}{\left(S_{i}^{c}-{𝔼}_{S^{c}\sim p\left(S^{c}|G\right)}\left[S^{c}\right]\right)}^{2}. \end{array}$$

#### 3.2.2 Explanation uncertainty from structural uncertainty

**Figure 1:**
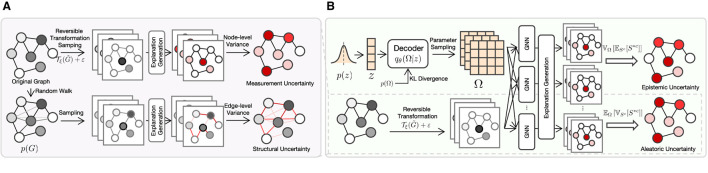
The overall structure of EU-GNN. **(A)** Quantifying explanation uncertainty from aleatoric uncertainty. We obtain data through reversible transformation and sampling from the edge distribution, respectively, calculate the saliency map and quantify the measurement uncertainty and structure uncertainty through variance. **(B)** Decomposing explanation uncertainty. By learning the parameter distribution from the data, we compute the total explanation uncertainty and decouple it into aleatoric uncertainty and epistemic uncertainty.

According to Bojchevski et al. (2018); Zhang et al. (2021); Ling et al. (2021, 2023b), the structural distribution of a graph *p*(*G*) can be approximated by the stationary distribution of random walks on the graph. In this work, we utilize random walks to capture the structural uncertainty. Suppose we could sample many graph instances and corresponding labels {*G*_*i*_ = (*V*_*i*_, *E*_*i*_, *X*_*i*_, *A*_*i*_), *y*_*i*_}, *i*∈{1, ⋯ , *I*} from the same distribution *p*(*G*). For each graph, we could calculate the saliency map $S_{i}^{c}$ of each sampled graph with $S_{i}^{c}=g\left(X_{i},A_{i},\Omega \right)$. The variance of explanation caused by the structural uncertainty can then be observed by:


$$\begin{array}{l} \frac{1}{I-1}\sum_{i=1}^{I}{\left(S_{i}^{c}-{\bar{S}}^{c}\right)}^{2}, \end{array}$$

where ${\bar{S}}^{c}=\frac{1}{I}\overset{I}{\underset{i=1}{\sum }}S_{i}^{c}$. Both types of aleatoric uncertainties are illustrated in Figure 1A. We first generate graphs from the original graph via reversible transformation sampling and random walk sampling, respectively, then feed them to the explanation generation model to get saliency maps. Finally, the measurement and structural uncertainties are quantified by computing the node-level and edge-level variance of the saliency maps, respectively.

### 3.3 Variational inference-based explanation uncertainty quantification

Other than aleatoric uncertainties, the explanation is also sensitive to epistemic uncertainty, which is typically caused by the mismatching between the distribution of learned model parameters and the correct underlying distribution. In this subsection, we consider quantifying both types of explanation uncertainties jointly. Specifically, we will elaborate on quantifying the total explanation uncertainty and further decomposing it into aleatoric and epistemic uncertainties.

Let {G,Y} denotes a training dataset, where G=(V,E,X,A)= ${\left\{\left(V_{t},{𝔼}_{t},X_{t},A_{t}\right)\right\}}_{t=1}^{T}=$ ${\left\{G_{t}\right\}}_{t=1}^{T}$ represents the set of *T* training inputs, and $Y={\left\{y_{t}\right\}}_{t=1}^{T}$ are the corresponding graph classification labels. We aim to obtain the posterior distribution of the model parameter p(Ω|X,A,Y), such that given a test sample (X^*^, A^*^), the classification label of can be predicted by:


$$\begin{array}{l} \;p\left(y^{*}|X^{*},A^{*},X,A,Y\right)=\int p\left(y^{*}|X^{*},A^{*},\Omega \right)p\left(\Omega |X,A,Y\right)\;d\;\Omega \\ =∭ℱ\left(T_{\xi }^{-1}\left(X^{*}-\varepsilon \right),A^{*},\Omega \right)p\left(\Omega |X,A,Y\right)p\left(\varepsilon \right)p\left(\xi \right)d\Omega d\xi d\varepsilon . \end{array}$$

Because the posterior p(Ω|X,A,Y) in the above function is intractable, it is further approximated with a variational distribution *q*_θ_(Ω|*z*), which is implemented as a decoder responsible for decoding the network parameter Ω from *z*. In this context, *z* is a sample drawn from a standard Gaussian distribution, denoted as p(z)=N(0,I).

Optimizing the decoder can be accomplished by minimizing the Kullback-Leibler divergence (KL-divergence) between the approximated posterior *E*_*z*_[*q*_θ_(Ω|*z*)] = ∫*p*(*z*)*q*_θ_(Ω|*z*)*dz* and the true posterior p(Ω|X,A,Y), which is defined as:


(3) $$\begin{array}{l} KL[{𝔼}_{z}[q_{\theta }\left(\Omega |z\right)]\|p\left(\Omega |X,A,Y\right)]={𝔼}_{\Omega \sim {𝔼}_{z}[q_{\theta }\left(\Omega |z\right)]}\log{𝔼}_{z}[q_{\theta }\left(\Omega |z\right)]\\ -{𝔼}_{\Omega \sim {𝔼}_{z}[q_{\theta }\left(\Omega |z\right)]}\log p\left(\Omega |X,A,Y\right). \end{array}$$

Note that Equation (3) is still intractable due to the unknown p(Ω|X,A,Y). To address this problem, we rewrite the KL-divergence as follows:


(4) $$\begin{array}{l} KL[{𝔼}_{z}[q_{\theta }\left(\Omega |z\right)]\|p\left(\Omega |X,A,Y\right)]\\ ={𝔼}_{\Omega }\log{𝔼}_{z}[q_{\theta }\left(\Omega |z\right)]-{𝔼}_{\Omega }\log\frac{p\left(\Omega \right)p\left(Y|X,A,\Omega \right)}{p\left(Y|X,A\right)}\\ =\log p\left(Y|X,A\right)-\left(-{𝔼}_{\Omega }\log\frac{{𝔼}_{z}[q_{\theta }\left(\Omega |z\right)]}{p\left(\Omega \right)}+{𝔼}_{\Omega }\log p\left(Y|X,A,\Omega \right)\right)\\ =\log p\left(Y|X,A\right)-(-KL[{𝔼}_{z}[q_{\theta }\left(\Omega |z\right)]\|p\left(\Omega \right)]+{𝔼}_{\Omega }\log p\left(Y|X,A,\Omega \right))\\ =\log p\left(Y|X,A\right)-(-KL[{𝔼}_{z}[q_{\theta }\left(\Omega |z\right)]\|p\left(\Omega \right)]\\ +{𝔼}_{\Omega }\left[\sum_{t=1}^{T}\log ℱ\left(T_{\xi }^{-1}\left(X_{t}-\varepsilon \right),A_{t},\Omega \right)\right]), \end{array}$$

where *p*(Ω) is the prior distribution for Ω, and For the sake of simplicity, we omit the subscript of *E*_Ω~_*E*__*z*_[*q*_θ_(Ω|*z*)]_ as *E*_Ω_ when the context is clear. Since the first term in Equation (4) (i.e., the evidence) is constant w.r.t θ, minimizing Equation (3) is equivalent to maximizing the second term, i.e., Evidence Lower Bound (ELBO). By introducing a weighting factor γ>0 to the ELBO in Equation (4), we define the loss function $L_{e}$ as:


(5) $$\begin{array}{l} {ℒ}_{e}=\gamma \cdot KL\left[{𝔼}_{z}[q_{\theta }\left(\Omega |z\right)]\|p\left(\Omega \right)\right]\\ \;-{𝔼}_{\Omega \sim {𝔼}_{z}[q_{\theta }\left(\Omega |z\right)]}\left[\sum_{t=1}^{T}\log ℱ\left(T_{\xi }^{-1}\left(X_{t}-\varepsilon \right),A_{t},\Omega \right)\right]. \end{array}$$

The $L_{e}$ is Equation (6) can further be approximated by:


(6) $$\begin{array}{l} p\left(y^{*}|X^{*},A^{*},X,A,Y\right)\\ =\oint ℱ\left(T_{\xi }^{-1}\left(X^{*}-\varepsilon \right),A^{*},\Omega \right)p\left(\Omega |X,A,Y\right)p\left(\varepsilon \right)p\left(\xi \right)d\Omega d\xi d\varepsilon \\ \approx \oint ℱ\left(T_{\xi }^{-1}\left(X^{*}-\varepsilon \right),A^{*},\Omega \right)p(z)q_{\theta }\left(\Omega |z\right)p(\xi )p(\varepsilon )dzd\Omega d\xi d\varepsilon \\ \approx \frac{1}{J\cdot K}\sum_{j=1}^{J}\sum_{k=1}^{K}ℱ\left(T_{\xi_{k}}^{-1}\left(X^{*}-\varepsilon_{k}\right),A^{*},\Omega_{j}\right). \end{array}$$

Similarly, we can obtain the saliency map from the following distribution:


(7) $$\begin{array}{l} p\left(S^{*c}|X^{*},A^{*},X,A,Y\right)\\ \;=\oint T_{\xi }\left(g\left(T_{\xi }^{-1}\left(X^{*}-\varepsilon \right),A^{*},\Omega \right)\right)p\left(\Omega |X,A,Y\right)p\left(\varepsilon \right)p\left(\xi \right)d\Omega d\xi d\varepsilon \\ \;\approx \frac{1}{J\cdot K}\sum_{j=1}^{J}\sum_{k=1}^{K}T_{\xi_{k}}\left(g\left(T_{\xi_{k}}^{-1}\left(X^{*}-\varepsilon_{k}\right),A^{*},\Omega_{j}\right)\right), \end{array}$$

where in Equations (7) and (8) ξ_*k*_~*p*(ξ), ε_*k*_~*p*(ε), *z*_*i*_~*p*(*z*), and Ω_*j*_~*q*_θ_(Ω|*z*_*i*_) are Monte Carlo simulation samples. Finally, the total explanation uncertainty is quantified by the variance of the saliency map S^*c^ under the probability distribution described in Equation (7), i.e., $V_{S^{*c}\sim p\left(S^{*c}|X^{*},A^{*},X,A,Y\right)}\left[S^{*c}\right]$. In the subsequent lemma, we show that the explanation uncertainty can be decomposed into aleatoric and epistemic uncertainty.

** Lemma 1**. The explanation uncertainty can be decoupled into explanation uncertainty attributed to data and to the model, respectively:


(8) $$\begin{array}{l} V_{S^{*c}\sim p\left(S^{*c}|X^{*},A^{*},X,A,Y\right)}[S^{*c}]\\ \;={𝔼}_{\Omega \sim p\left(\Omega |X,A,Y\right)}\left[V_{S^{c}\sim p\left(S^{*c}|X^{*},A^{*},\Omega \right)}[S^{*c}]\right]\\ \;+V_{\Omega \sim p\left(\Omega |X,A,Y\right)}\left[{𝔼}_{S^{c}\sim p\left(S^{c}|X^{*},A^{*},\Omega \right)}[S^{*c}]\right]\\ \approx \underset{\text{Aleatoric Uncertainty}}{\underset{︸}{{𝔼}_{\Omega \sim {𝔼}_{z\sim q_{\theta }[q_{\phi }\left(\Omega |z\right)]}}\left[V_{S^{c}\sim p\left(S^{*c}|X^{*},A^{*},\Omega \right)}[S^{*c}]\right]}} \end{array}$$

(9) $$+\underset{\text{Epistemic Uncertainty}}{\underset{︸}{V_{\Omega \sim {𝔼}_{z\sim q_{\theta }[q_{\phi }\left(\Omega |z\right)]}}\left[{𝔼}_{S^{*c}\sim p\left(S^{*c}|X^{*},A^{*},\Omega \right)}[S^{*c}]\right]}}.$$

The proof for Lemma 1 is given as follows:

Proof. We first prove Equation (9).


(10) $$\begin{array}{l} {𝔼}_{\Omega \sim p\left(\Omega |X,A,Y\right)}\left[V_{S^{c}\sim p\left(S^{*c}|X^{*},A^{*},\Omega \right)}[S^{*c}]\right]\\ \;+V_{\Omega \sim p\left(\Omega |X,A,Y\right)}\left[{𝔼}_{S^{c}\sim p\left(S^{c}|X^{*},A^{*},\Omega \right)}[S^{*c}]\right]\\ \;={𝔼}_{\Omega \sim p\left(\Omega |X,A,Y\right)}[{𝔼}_{S^{c}\sim p\left(S^{*c}|X^{*},A^{*},\Omega \right)}\left[{\left(S^{*c}\right)}^{2}\right]\\ \;-{𝔼}_{S^{c}\sim p\left(S^{*c}|X^{*},A^{*},\Omega \right)}^{2}\left[S^{*c}\right]]+{𝔼}_{\Omega \sim p\left(\Omega |X,A,Y\right)}\left[{𝔼}_{S^{c}\sim p\left(S^{c}|X^{*},A^{*},\Omega \right)}^{2}[S^{*c}]\right]\\ \;-{𝔼}_{\Omega \sim p\left(\Omega |X,A,Y\right)}^{2}\left[{𝔼}_{S^{c}\sim p\left(S^{c}|X^{*},A^{*},\Omega \right)}[S^{*c}]\right]\\ \left(V_{x\sim p(x)}[x]={𝔼}_{x\sim p(x)}\left[x^{2}\right]-{𝔼}_{x\sim p(x)}^{2}[x]\right)\\ \;={𝔼}_{\Omega \sim p\left(\Omega |X,A,Y\right)}\left[{𝔼}_{S^{c}\sim p\left(S^{*c}|X^{*},A^{*},\Omega \right)}\left[{\left(S^{*c}\right)}^{2}\right]\right]\\ \;-{𝔼}_{\Omega \sim p\left(\Omega |X,A,Y\right)}^{2}\left[{𝔼}_{S^{c}\sim p\left(S^{c}|X^{*},A^{*},\Omega \right)}[S^{*c}]\right]. \end{array}$$

Moreover,


(11) $$\begin{array}{l} {𝔼}_{\Omega \sim p(\Omega |X,A,Y)}\left[{𝔼}_{S^{*c}\sim p\left(S^{*c}|X^{*},A^{*},\Omega \right)}\left[{\left(S^{*c}\right)}^{2}\right]\right]\\ \;=\int p(\Omega |X,A,Y)\int {\left(S^{*c}\right)}^{2}p\left(S^{*c}|X^{*},A^{*},\Omega \right)dS^{*c}d\Omega \\ \;=\int p(\Omega |X,A,Y)\int {\left(S^{*c}\right)}^{2}p\left(S^{*c}|X^{*},A^{*},X,A,Y,\Omega \right)dS^{*c}d\Omega \\ \left(S^{*c}\text{ and }(X,A,Y)\text{ are independent given }\Omega \right)\\ \;=\iint {\left(S^{*c}\right)}^{2}p\left(S^{*c},\Omega |X^{*},A^{*},X,A,Y\right)dS^{*c}d\Omega \\ \;=\int {\left(S^{*c}\right)}^{2}p\left(S^{*c}|X^{*},A^{*},X,A,Y\right)dS^{*c}\\ \;={𝔼}_{S^{*c}\sim p\left(S^{*c}|X^{*},A^{*},X,A,Y\right)}\left[{\left(S^{*c}\right)}^{2}\right]. \end{array}$$

Plugging Equation (14) into Equation (11), we have:


$$\begin{array}{l} {𝔼}_{\Omega \sim p\left(\Omega |X,A,Y\right)}\left[{𝔼}_{S^{c}\sim p\left(S^{*c}|X^{*},A^{*},\Omega \right)}\left[{\left(S^{*c}\right)}^{2}\right]\right]\\ \;-{𝔼}_{\Omega \sim p\left(\Omega |X,A,Y\right)}^{2}\left[{𝔼}_{S^{c}\sim p\left(S^{c}|X^{*},A^{*},\Omega \right)}[S^{*c}]\right]\\ \;={𝔼}_{S^{*c}\sim p\left(S^{*c}|X^{*},A^{*},X,A,Y\right)}\left[{\left(S^{*c}\right)}^{2}\right]\\ \;-{𝔼}_{\Omega \sim p\left(\Omega |X,A,Y\right)}^{2}\left[{𝔼}_{S^{c}\sim p\left(S^{c}|X^{*},A^{*},\Omega \right)}[S^{*c}]\right]\\ \;=V_{S^{*c}\sim p\left(S^{*c}|X^{*},A^{*},X,A,Y\right)}[S^{*c}]. \end{array}$$

Hence, Equation (9) is proved. Finally, by approximating p(Ω|X,A,Y) with *E*_*z*~_*q*__θ_[*q*_ϕ_(Ω|*z*)]_, Equation (10) is proved.

The explanation uncertainty decomposition is depicted in Figure 1B. Generally, the top and down portions of Figure 1B portray the estimation of epistemic and aleatoric uncertainties, respectively. As shown in the top portion, we first sample an *z* from a prior distribution *p*(*z*) that is then fed to the Decoder jointly with the prior distribution of the GNN model parameter *p*(Ω) to output the estimated posterior distribution *q*_θ_(Ω|*z*). Next, we sample a group of Ωs in *q*_θ_(Ω|*z*) to get a group of GNN models, which further generate a group of explanations. The lower portion of Figure 1B is the same as Figure 1A. Finally, the epistemic and aleatoric uncertainties are computed with the guide of Lemma 1.

## 4 Experiment

In this section, we aim to establish a clear link between methodological advancements and experimental validations. Our experiments demonstrate that EU-GNN not only advances the state-of-the-art in GNN explanation performance (Section 4.2) but also provides a robust measure of uncertainty (Sections 4.3 and 4.4), leading to more trustworthy and interpretable GNN models. We show the results of quantitative and qualitative experiments that were performed to evaluate our method with other comparison models. Three real-world graph classification datasets are introduced in our experiments. The experiments in this paper were performed on a 64-bit machine with 14-core Intel Xeon(R) Gold 6330, 80 GB memory, and NVIDIA RTX 3090 GPU.

### 4.1 Experimental setup

#### 4.1.1 Datasets

We investigate three binary classification molecular datasets: BBBP (Martins et al., 2012), BACE (Subramanian et al., 2016), and TOX21 (Mayr et al., 2016)^1^, focusing on the identification of functional groups in organic molecules related to biological molecular properties. Each dataset comprises experimentally determined binary classifications of small organic molecules. All three datasets are divided into training, validation, and testing sets with a ratio of 8:1:1. the scaffold split method is employed for both BBBP and BACE datasets, grouping molecules with similar structures within the same division. Conversely, the TOX21 dataset utilizes a random splitting approach. The detailed information for these datasets is as follows:

- **BBBP:** The Blood-brain barrier penetration (BBBP) dataset comes from a recent study (Martins et al., 2012) on the modeling and prediction of barrier permeability. As a membrane separating circulating blood and brain extracellular fluid, the blood-brain barrier blocks most drugs, hormones, and neurotransmitters. Thus, penetration of the barrier forms a long-standing issue in the development of drugs targeting the central nervous system. This dataset includes binary labels for 2,053 compounds (graphs) on their permeability properties.
- **BACE:** The BACE dataset provides quantitative (IC50) and qualitative (binary label) binding results for a set of inhibitors of human b-secretase 1 (BACE-1) (Subramanian et al., 2016). This dataset contains a collection of 1,522 compounds (graphs) with their 2D structures and binary labels.
- **TOX21:** The “Toxicology in the 21st Century” (TOX21) initiative created a public database measuring the toxicity of compounds. The original dataset contains qualitative toxicity measurements for 8,014 compounds (graphs) on 12 different tasks, here we selected the NR-ER task, which is concerned with the activation of the estrogen receptor (Mayr et al., 2016).

#### 4.1.2 Compasion methods

In this work, we analyze the uncertainty of explanation generated in the *graph classification* task. We analyze the explanation uncertainty with respect to both aleatoric uncertainty and epistemic uncertainty with two categories of methods:

- *General GNNs that do not consider quantifying uncertainties:* (1) GCN (Kipf and Welling, 2016) is the classical graph neural networks that specialize in learning representations and patterns in graph-structured data, (2) GAT (Veličković et al., 2017) leverages attention mechanisms to weigh and aggregate neighboring node information effectively, (3) GIN (Xu et al., 2018) is a powerful graph neural network architecture designed to capture intricate structural information by considering node and edge attributes for graph representation learning.
- *GNNs with uncertainty quantification mechanisms*: (1) Bayesian GCN (Pal et al., 2019) is a novel approach that combines Bayesian inference and non-parametric graph learning techniques to improve the robustness and interpretability of GNNs, (2) GDC (Hasanzadeh et al., 2020) proposes a unified framework for adaptive connection sampling that generalizes existing stochastic regularization methods for training GNNs, (3) BGNN-AE (Munikoti et al., 2023) proposes a unified framework to measure the aleatoric and epistemic uncertainty for GNNs.

#### 4.1.3 Implementation details

In this paper, our method is implemented in PyTorch. The decoder network is a three-layer feed-forward network (FNN) with a hidden size of 512, 256, 128, and the Sigmoid activation function. The mean operator is utilized as the readout function and the activation function is Softmax. For optimization, we use the Adam optimizer (Kingma and Ba, 2014) with a learning rate of 0.001 for all the baselines. The node-level explanation is calculated by the Grad-CAM formulation, and the edge-level explanation is specified following the gradient-based formulation in Gao et al. (2021). All experiments are repeated 10 times for each method, and we report the average results and the standard deviation in the following quantitative analysis.

#### 4.1.4 Evaluation metrics

Since we aim at the general graph classification problem. To this end, we introduce three fundamental classification metrics, carefully chosen to evaluate the methods in a comprehensive manner: (1). Accuracy (ACC): ACC serves as an intuitive performance metric to assess a model's effectiveness in distinguishing different graph structures. (2). F1 score (F1): Given the potential class imbalance in graph classification tasks, the F1 score, as a harmonic mean of precision and recall, offers a more balanced evaluation of a model's ability to correctly classify graphs while minimizing both false positives and false negatives. (3). Area Under Curve (AUC): AUC quantifies a model's discriminative power between different graph classes, with higher AUC values indicating superior classification performance irrespective of varying class distributions.

### 4.2 Graph classification performance analysis

To make a trustworthy prediction explanation, the first step is to analyze the prediction accuracy. Following the experiment setup in Munikoti et al. (2023), we manually add white noise (i.e., aleatoric uncertainty) to the test data and see how each model resists the perturbation. Specifically, we add noise (sampled from Gaussian Distribution) randomly to a portion of nodes' features (i.e., 0, 5, 10%) and demonstrate the performance comparison on three molecule classification datasets.

According to Table 1, our proposed EU-GNN constantly achieves the best performance among all GNN models in three molecule datasets. To be more specific, without any perturbations (0%), our proposed EU-GNN can already surpass other methods. For example, EU-GNN achieves the best results in all three datasets by excelling other baselines on the most 1.8% in the BACE dataset. With gradually adding perturbations, our method still achieves the best classification accuracy and guarantees robustness. Notably, Compared with the optimal baseline, EU-GNN achieves the improvement of 5.8 and 3.9% under 5 and 10% perturbation, respectively. The variance of 10 rounds of experiments on all datasets is <0.002. The performance achievement is mainly due to the unified uncertainty measurement and data-based parameter generation reducing the risk of overfitting. An interesting finding is that state-of-the-art GNN with UQ, BGCN, and BGNN-AE do not always outperform GIN in low-noise conditions. This may benefit from the ability of GIN to extract graph structure information, while the introduction of randomness affects the accuracy of BCGN and BGNN-AE.

**Table 1:** Graph classification performance comparison with white noise.

**Method**	**0%**			**5%**			**10%**		

	**ACC**	**AUC**	**F1**	**ACC**	**AUC**	**F1**	**ACC**	**AUC**	**F1**
**BBBP**									
GCN	0.658 (0.0056)	0.612 (0.0047)	0.584 (0.0046)	0.581 (0.0092)	0.534 (0.0087)	0.515 (0.0084)	0.548 (0.0114)	0.503 (0.0108)	0.48 (0.0105)
GAT	0.656 (0.0051)	0.604 (0.0054)	0.578 (0.0042)	0.596 (0.0082)	0.525 (0.0082)	0.511 (0.008)	0.549 (0.0106)	0.503 (0.0114)	0.478 (0.0112)
GIN	0.661 (0.0047)	0.614 (0.005)	0.586 (0.0046)	0.582 (0.0088)	0.539 (0.0084)	0.523 (0.0084)	0.558 (0.0111)	0.506 (0.0103)	0.479 (0.0099)
Bayesian GCN	0.659 (0.0033)	0.613 (0.0031)	0.584 (0.0021)	0.604 (0.0048)	0.555 (0.005)	0.526 (0.0058)	0.56 (0.0076)	0.525 (0.0073)	0.486 (0.0069)
BGNN-AE	0.659 (0.0029)	0.613 (0.0034)	0.584 (0.0025)	0.599 (0.0054)	0.554 (0.0052)	0.529 (0.0047)	0.566 (0.0071)	0.529 (0.0072)	0.488 (0.0075)
GDC	0.662 (0.0026)	0.614 (0.0034)	0.587 (0.0032)	0.601 (0.0052)	0.553 (0.0042)	0.528 (0.0046)	0.554 (0.0058)	0.527 (0.0051)	0.483 (0.0054)
**EU-GNN**	**0.665 (0.0012)**	**0.619 (0.0016)**	**0.589 (0.0011)**	**0.615 (0.0027)**	**0.576 (0.0033)**	**0.542 (0.003)**	**0.592 (0.0042)**	**0.536 (0.0043)**	**0.51 (0.0046)**
**BACE**									
GCN	0.704 (0.0054)	0.653 (0.0059)	0.615 (0.005)	0.632 (0.0084)	0.583 (0.0076)	0.525 (0.0087)	0.604 (0.0102)	0.543 (0.0106)	0.5 (0.0107)
GAT	0.708 (0.0055)	0.655 (0.0045)	0.616 (0.0057)	0.631 (0.0089)	0.575 (0.0089)	0.545 (0.0081)	0.603 (0.0108)	0.548 (0.011)	0.497 (0.01)
GIN	0.712 (0.0044)	0.659 (0.0049)	0.62 (0.0042)	0.626 (0.0094)	0.588 (0.0084)	0.536 (0.0089)	0.606 (0.0106)	0.55 (0.0113)	0.504 (0.0113)
Bayesian GCN	0.709 (0.003)	0.655 (0.0032)	0.617 (0.0022)	0.642 (0.0059)	0.586 (0.0058)	0.553 (0.0057)	0.629 (0.0076)	0.566 (0.0071)	0.531 (0.0079)
BGNN-AE	0.713 (0.0034)	0.66 (0.0027)	0.621 (0.0034)	0.642 (0.005)	0.596 (0.0045)	0.558 (0.0057)	0.628 (0.0077)	0.572 (0.008)	0.528 (0.0078)
GDC	0.712 (0.0018)	0.661 (0.0022)	0.619 (0.0028)	0.656 (0.0055)	0.611 (0.0052)	0.562 (0.0047)	0.641 (0.0049)	0.582 (0.0056)	0.535 (0.0053)
**EU-GNN**	**0.726 (0.0014)**	**0.664 (0.0018)**	**0.624 (0.0018)**	**0.681 (0.0033)**	**0.623 (0.0033)**	**0.575 (0.0032)**	**0.651 (0.0046)**	**0.593 (0.0047)**	**0.548 (0.0042)**
**TOX 21**									
GCN	0.788 (0.0048)	0.744 (0.0048)	0.707 (0.0058)	0.699 (0.0086)	0.658 (0.0081)	0.618 (0.0082)	0.676 (0.0106)	0.635 (0.0109)	0.601 (0.011)
GAT	0.786 (0.005)	0.743 (0.0041)	0.707 (0.0044)	0.699 (0.0092)	0.663 (0.0083)	0.631 (0.0081)	0.686 (0.0109)	0.636 (0.0098)	0.597 (0.0112)
GIN	0.791 (0.0041)	0.747 (0.0051)	0.711 (0.004)	0.704 (0.0084)	0.663 (0.009)	0.639 (0.0078)	0.686 (0.0105)	0.637 (0.0096)	0.607 (0.0103)
Bayesian GCN	0.788 (0.0024)	0.744 (0.0029)	0.707 (0.0026)	0.721 (0.0048)	0.673 (0.0047)	0.635 (0.0046)	0.707 (0.0072)	0.647 (0.007)	0.621 (0.0069)
BGNN-AE	0.789 (0.004)	0.745 (0.0034)	0.708 (0.0025)	0.717 (0.0046)	0.681 (0.0057)	0.638 (0.0054)	0.706 (0.0079)	0.658 (0.0072)	0.62 (0.0072)
GDC	0.789 (0.0021)	0.749 (0.0032)	0.714 (0.0031)	0.724 (0.0053)	0.693 (0.0046)	0.654 (0.0048)	0.712 (0.005)	0.664 (0.0053)	0.632 (0.005)
**EU-GNN**	**0.793 (0.0017)**	**0.758 (0.0027)**	**0.718 (0.0008)**	**0.745 (0.0034)**	**0.711 (0.0035)**	**0.669 (0.0032)**	**0.716 (0.0039)**	**0.674 (0.0044)**	**0.641 (0.0039)**

The results are obtained from 10 individual runs for every setting. The best is highlighted in bold. The percentage means the rate of noise added to the dataset.

### 4.3 Explanation uncertainty measurement

**Figure 2:**
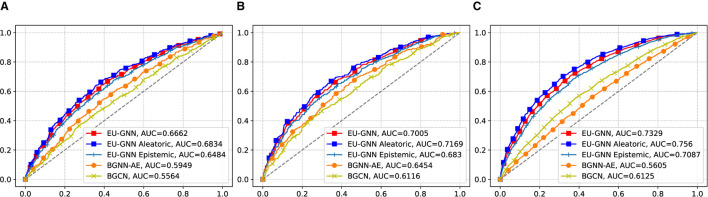
The misclassification performance on graph classification. The *x*-axis represents the False Positive Rate, and the *y*-axis represents the True Positive Rate. **(A)** BBBP. **(B)** BACE. **(C)** TOX 21.

To evaluate the quality of the measured uncertainty, we conduct the misclassification detection experiment. Specifically, the misclassification detection experiment involves detecting whether a given prediction is incorrect using an uncertainty estimate. The misclassification detection experiment is used as an application to test the performance of our method. Intuitively, if the explanation of the prediction is wrong, our model should give a relatively higher uncertainty score. Conversely, our model should give a lower uncertainty score for those correctly classified samples. We regard all misclassified samples as positive samples, use the uncertainty score output by the model as the score and draw the ROC curve. Figure 2 shows that our EU-GNN outperforms other UQ for GNN methods with improvements of 11.9, 8.5, and 19.7% on BBBP, BACE, and TOX21 datasets, respectively. The results prove that for misclassified samples, our method has a good recognition ability based on uncertainty measurement. Further, we decouple the total uncertainty into aleatoric uncertainty and epistemic uncertainty. It is clear that both aleatoric uncertainty and epistemic uncertainty exhibit excellent misclassification detection capabilities. Moreover, we observe that the performance of aleatoric uncertainty is better, which indicates the importance of conducting explanation uncertainty based on aleatoric uncertainty. Such a discerning distinction in uncertainties facilitates an enriched understanding, aiding in not only enhancing the predictive model but also in providing a potential for explanation analysis.

### 4.4 Uncertainty explanation performance

As depicted in Table 2, we employ the public human-annotated explanations for the BBBP dataset from Gao et al. (2021) to assess the explanation uncertainty of our model. Specifically, for each molecule, we take the mean value of the calculated node-level and edge-level saliency maps. We then normalize these values to a 0–1 range and use 0.5 as the threshold for explanation classification. Subsequently, we perform explanation misclassification detection leveraging both measurement and structural uncertainties. For BGCN and BGNN-AE, we directly employ the Grad-CAM output on the node and edge feature maps and compute the variance to represent uncertainty. The results indicate that EU-GNN provides a more accurate and consistent performance in explanation misclassification detection compared to other UQ for GNN methods, underscoring the effectiveness of our explanation uncertainty.

**Table 2:** Explanation misclassification detection performance on BBBP dataset.

**Method**	**Node ACC**	**Node AUC**	**Edge ACC**	**Edge AUC**
BGCN	0.715 (0.0033)	0.678 (0.0041)	0.844 (0.0027)	0.803 (0.0036)
BGNN-AE	0.722 (0.0043)	0.687 (0.0045)	0.852 (0.0038)	0.825 (0.0035)
**EU-GNN**	**0.746 (0.0015)**	**0.703 (0.0017)**	**0.877 (0.0017)**	**0.846 (0.0021)**

The results are obtained from 10 individual runs for every setting. The best is highlighted in bold. The percentage means the rate of noise added to the dataset.

### 4.5 Ablation study

We further conduct the ablation study to investigate the importance of the proposed components of EU-GNN. We consider two variants: (1) EU-GNN-NT represents EU-GNN without reversible transformation. (2) EU-GNN-NV removes the Decoder and directly optimizes the parameters during training. The results shown in Table 3 prove the necessity of our proposed components. Particularly, EU-GNN-NT outperforms its counterpart albeit exhibiting a tangible limitation primarily attributed to its inability to accurately capture data fluctuations, which ostensibly hampers its predictive prowess in scenarios characterized by volatile data points. Moreover, EU-GNN-NV, even though it degenerates into a conventional GCN post the Decoder's removal, the intrinsic incorporation of the reversible transformation fortuitously ensures it maintains a commendable performance, especially when confronted with datasets suffused with high noise levels. Thus, it outperforms the benchmark GCN under, further corroborating the imperative role of the reversible transformation in safeguarding model robustness and enhancing performance efficacy.

**Table 3:** Ablation study for EU-GNN and the variants.

**Method**	**0%**			**5%**			**10%**		

	**ACC**	**AUC**	**F1**	**ACC**	**AUC**	**F1**	**ACC**	**AUC**	**F1**
**BBBP**									
EU-GNN-NT	0.662 (0.0022)	0.616 (0.0031)	0.588 (0.002)	0.607 (0.0043)	0.566 (0.0038)	0.537 (0.0037)	0.586 (0.0048)	0.529 (0.0045)	0.503 (0.0055)
EU-GNN-NV	0.658 (0.0025)	0.611 (0.0031)	0.583 (0.0027)	0.585 (0.0039)	0.537 (0.0042)	0.514 (0.0044)	0.556 (0.051)	0.511 (0.0053)	0.485 (0.0051)
**EU-GNN**	**0.665 (0.0012)**	**0.619 (0.0016)**	**0.589 (0.0011)**	**0.615 (0.0027)**	**0.576 (0.0033)**	**0.542 (0.003)**	**0.592 (0.0042)**	**0.536 (0.0043)**	**0.51 (0.0046)**
**BACE**									
EU-GNN-NT	0.718 (0.0024)	0.616 (0.0029)	0.621 (0.0023)	0.674 (0.0035)	0.617 (0.0035)	0.568 (0.0037)	0.648 (0.0043)	0.59 (0.0047)	0.544 (0.0047)
EU-GNN-NV	0.706 (0.0024)	0.654 (0.0032)	0.617 (0.0031)	0.646 (0.0042)	0.597 (0.0042)	0.536 (0.0047)	0.621 (0.0056)	0.561 (0.0053)	0.512 (0.0059)
**EU-GNN**	**0.726 (0.0014)**	**0.664 (0.0018)**	**0.624 (0.0018)**	**0.681 (0.0033)**	**0.623 (0.0033)**	**0.575 (0.0032)**	**0.651 (0.0046)**	**0.593 (0.0047)**	**0.548 (0.0042)**
**TOX 21**									
EU-GNN-NT	0.789 (0.0019)	0.751 (0.0031)	0.732 (0.0019)	0.737 (0.0038)	0.702 (0.0035)	0.659 (0.0041)	0.715 (0.0049)	0.671 (0.0047)	0.636 (0.0052)
EU-GNN-NV	0.789 (0.0026)	0.75 (0.0035)	0.732 (0.0034)	0.706 (0.0044)	0.667 (0.0041)	0.642 (0.0036)	0.693 (0.0052)	0.641 (0.0054)	0.615 (0.0054)
**EU-GNN**	**0.793 (0.0017)**	**0.758 (0.0027)**	**0.718 (0.0008)**	**0.745 (0.0034)**	**0.711 (0.0035)**	**0.669 (0.0032)**	**0.716 (0.0039)**	**0.674 (0.0044)**	**0.641 (0.0039)**

The results are obtained from 10 individual runs for every setting. The best is highlighted in bold. The percentage means the rate of noise added to the dataset.

### 4.6 Visualization

**Figure 3:**
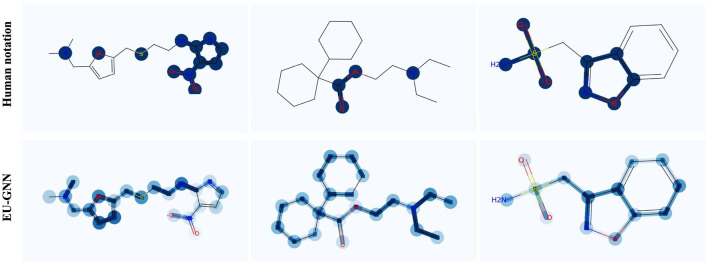
Visualization of explanation uncertainty on molecule samples. For human notation, dark nodes and edges represent important explanations. For EU-GNN, deeper nodes and edges represent greater uncertainty.

Finally, we visualize three samples to demonstrate the effectiveness of both measurement uncertainty and structural uncertainty in detecting misclassification explanations. As depicted in Figure 3, it's evident that our explanation uncertainty effectively identifies incorrect explanations. Taking a deeper dive into the specificities, consider sample 1: the measurement uncertainty notably brings to light an erroneous identification, wherein two carbon atoms situated on the furan were mistakenly recognized as valid structures with regard to barrier permeability. Furthermore, the structural uncertainty embedded within EU-GNN comes to the fore in sample 3: it projects a heightened uncertainty for the furan and its interconnected edges. Notably, when juxtaposed with human annotations, none of these highlighted structures were deemed valid constructs, thereby underpinning the precision and applicability of our model in discerning and subsequently spotlighting the anomalies in classification explanations.

## 5 Conclusion

In this paper, we propose a novel framework called EU-GNN, which is the first to quantify uncertainties of explanation on graph classification problems. EU-GNN is a unified framework to calculate different uncertainties simultaneously, in which we add the measurement uncertainty and structure uncertainty designed for graph data. Through the parameter distribution learned from the data, EU-GNN achieves to provide accurate uncertainty measure without prior knowledge. Meanwhile, our method can incorporate any GNN explanation techniques to measure explanation uncertainty. Extensive experiments on real-world datasets demonstrate the effectiveness and robustness of EU-GNN. Besides, our method shows superiority in measuring the uncertainties of explanation.

Future work can explore the following three directions: (1) Future research can further explore the impact of different types of uncertainties on GNN interpretability. For example, model structure uncertainty and data quality uncertainty, and study how they individually or jointly affect the interpretability of GNN. (2) Future research can focus on reducing the uncertainty faced in the GNN interpretation process. This may be achieved by creating new explanatory models that are able to minimize the impact of uncertainty while maintaining high interpretability. (3) Consider extending the EU-GNN framework to node classification tasks and large-scale graphs while maintaining accurate quantification of uncertainty.

## Data availability statement

Publicly available datasets were analyzed in this study. This data can be found here: https://moleculenet.org/datasets-1.

## Author contributions

JJ: Data curation, Methodology, Validation, Visualization, Writing – original draft. CL: Conceptualization, Methodology, Validation, Writing – original draft. HL: Conceptualization, Formal analysis, Methodology, Writing – original draft. GB: Conceptualization, Formal analysis, Methodology, Writing – original draft. XZ: Conceptualization, Investigation, Methodology, Validation, Writing – review & editing. LZ: Conceptualization, Funding acquisition, Project administration, Resources, Supervision, Writing – review & editing.

## Footnotes

^1^Available online at https://moleculenet.org/datasets-1
